# Richmond Academy of Medicine and Surgery

**Published:** 1897-12

**Authors:** Mark W. Peyser

**Affiliations:** Secretary and Reporter; Richmond, Va.


### RICHMOND ACADEMY OF MEDICINE AND SURGERY.

RICHMOND, VA., October 26, 1897.

First Vice-President, Dr. Paulus A. Irving, in the chair.

Dr. Jacob Michaux reported "A Case of Typhoid Fever, Relapsing, with Unusual Absence of Cerebral Manifestations, Resulting in Death." It began with the usual symptoms, running on three or four weeks with nothing abnormal. About the beginning of the fifth week there was a fall of temperature and convalescence began. A week after, there were decided evidences of intestinal disturbance from an unknown cause, but he suspected imprudence in diet. The patient rapidly grew worse, but (and this was an especial point of interest) there was a singular absence of cerebral symptoms. During the first part of the sickness she complained of the fear of losing her mind, but there was no delirium, although there might have been a little incoherence in going to, or awaking from, sleep, which was much less than he had ever seen in a severe case of typhoid. In the relapse there was a tendency to cerebral complications, the patient being occasionally unable to find the correct word, but she was conscious of it and embarrassed thereby, differing thus from the usual mental symptoms of typhoid. Dr. Michaux explained this absence: He had adopted the habit of allowing the bowels to run instead of checking them, believing that the detritus was gotten rid of and autointoxication avoided. In this case there were five or six stools in twenty-four hours, which he allowed to go on until there was complaint of weakness, when he used opium and lead, which acted well. This was during the first attack. During the relapse the bowel symptoms were marked and serious. The operations were allowed to continue and the following prescription, which seemed to produce no increase of the diarrhea, given every four hours: Calomel, one-twelfth grain; bicarbonate of sodium, one grain; and ipecac, one-twelfth grain. This was for asepsis and to stimulate the liver and eliminate the poison. He believed the unrestrained condition of the bowels accounted for the absence of cerebral symptoms and, for the same reason, there was little subsultus, although a slight amount of trembling was present when the patient was very sick. It might be argued, and with some reason, that while the course pursued relieved the system of detritus, it induced inflammation. Yet, when Epsom salt was given in acute dysentery the congested parts and the tendency to inflammation were relieved. He was not prepared to condemn *in toto* the Woodbridge treatment, but would not employ it indiscriminately.

Dr. Virginius W. Harrison reported a case of "Typhoid Ulceration and Perforation, with Operation." Youth, aged eighteen years, no previous sickness. He had progressed well up to the twenty-four hours before Dr. Harrison saw him in consultation. On Friday,

22d, he was attacked by a severe, persistent abdominal pain. Without advice, the father administered a large dose of whiskey. Early the next morning, 23d, the attending physician was sent for. Turpentine stupes were applied without relief. That night, two doses of Epsom salt were given, another early the next morning, 24th. No operation resulting, castor-oil was administered and late in the morning there was one. That afternoon Dr. Harrison was called in consultation and diagnosed perforating ulcer with septic peritonitis; prognosis, inevitable death without operation, which he advised. Consent being given, the patient was moved to Virginia Hospital, which he reached at 8:30 P.M. and the operation was done at 10 P.M. Incision was made in the median line and the perforation, which was easily found, was ascertained to be about the size of a goose quill and in the outer side of the ileum. Fecal matter was oozing from it. The abdominal cavity was full of pus and other fluid; the bowels filled with gas; and suppurative peritonitis was general. The edges of the perforation were trimmed and it was closed by three sets of sutures. The abdomen was irrigated with normal saline solution, several yards of gauze being inserted for drainage. The patient was put to bed in a condition better than that he possessed while on the table. Before the operation, one pint of normal saline solution was transfused venously, which improved the state. During the operation, strychnine was given hypodermically. The patient soon rallied, became conscious and conversed. In fifteen minutes the pulse began to fail rapidly and venous transfusion was resorted to. Improvement resulted, but the patient soon became so restless as to require holding in bed. He was called to the telephone, but before he could reach it, he was summoned back to find the patient dying.

Fifty-two operations for perforating typhoid ulcer have been reported, with 27 per cent. recovery, and the operation therefore is certainly worth the trial. The low condition of the patient is certainly no more a contraindication for operation than in suppurative peritonitis or pyosalpinx.

Dr. Mark W. Peyser reported an "Exaggerated Case of Urticaria with Syncope." Woman, white; married; aged forty years; history of gastric and intestinal indigestion, and tachycardia, which latter he believed functional. At dinner the patient had partaken of cabbage, had eaten little or no supper. Soon after dinner she felt an itching on her left hip and thinking she had been bitten by an insect, she investigated, but could see nothing except an elevated patch. Following, was a like sensation on the right hip, with like result, it spreading rapidly until the face, trunk and all the limbs were affected, and uticarious patches as large as the palm of the hand appeared. Receiving an urgent call that the woman was dying, the doctor hurried to her bedside to find her recovering from a profound syncope. The patches were intensely congested and stood in relief from the surface about an eighth of an inch, with edges puckered like scar tissue. Nitro-glycerine and strychnine were injected hypodermically, alkaline washes and enemata employed, and bromide of sodium given internally. Itching soon disappeared and the patient was up and about in three days. The only cause for the syncope that could be given was intense dilatation of the cutaneous vessels, depleting the heart and the internal circulation.

Dr. Paulus A. Irving reported a case of "Mechanical Intestinal Obstruction." The patient was found tossing in bed from excessive pain. The day before he had been perfectly well, had had a copious evacuation and ate a hearty supper. An hour after there were violent abdominal pains, which persisted the entire night in spite of various household remedies. The pain was localized around the umbilicus, and there was considerable gastric disturbance. Enemata brought away only a slight action, but the stomach became easier. Calomel was administered and followed by a saline, which came back. The patient was seen several times during the day, and at night was removed to Virginia Hospital. High enemata were given, but they came back only slightly stained. The patient gradually grew worse and was finally operated upon. Five or six inches above the ileo-cecal valve was found a band so tightly hugging the gut that when it was severed it made a popping noise. The patient reacted well, but because of peritonitis and paresis, no evacuation was had until the next evening and this was produced only after several high enemata. Recovery was uneventful. For a long time the patient had suffered from constipation and was much emaciated. It was hard to say what produced the inflammation. About ten years ago he had a similar attack, which must have been appendicitis, and the later trouble might have been due to it. The appendix was not carefully sought.

MARK W. PEYSER, M.D., Secretary and Reporter.

